# The efficacy and safety of weekly somatrogon treatment in turkish children with growth hormone deficiency: a real-world cohort study

**DOI:** 10.1007/s12020-026-04658-9

**Published:** 2026-05-21

**Authors:** Tahsin Gider, Edip Unal, Servet Yel, Barış Kolbaşı, Hüseyin Düzgün

**Affiliations:** 1 Dicle University Faculty of Medicine, Department of Pediatric Endocrinology, Diyarbakır, Turkey; 2 Dicle University Faculty of Medicine, Department of Pediatrics, Diyarbakır, Turkey

**Keywords:** Growth Hormone Deficiency, Somatrogon, Children

## Abstract

**Background:**

Long-acting growth hormone preparations have the potential to improve treatment compliance by reducing the frequency of injections. The aim of this study was to evaluate the efficacy and safety of weekly somatrogon treatment in patients with pediatric growth hormone deficiency (GHD) under real-world conditions.

**Methods:**

Patients with GHD aged 3–18 years who had been receiving somatrogon therapy for at least 6 months were included in the study. Anthropometric measurements, insulin-like growth factor-1 (IGF-1), bone age/chronological age (BA/CA) ratio, and drug-related adverse events were evaluated at baseline and at the final follow-up.

**Results:**

A total of 39 patients with a mean age of 12.90 ± 1.63 years were included. The mean treatment duration was 11.31 ± 2.72 months (min–max: 6.36–16.92). The mean annual height gain was 10.07 ± 0.61 cm, and the Δ height SDS was 0.64. No significant change was observed in the BA/CA ratio (*p* = 0.2). The mean baseline IGF-1 SDS was − 0.61 ± 1.15, increasing to 0.75 ± 1.26 at the final follow-up (*p* < 0.01). In 5 of 39 patients (12.8%), IGF-1 SDS exceeded + 2 SDS, requiring dose reduction. No serious adverse events were observed.

**Conclusion:**

Based on our real-world data, weekly somatrogon therapy provided an effective growth response and was well tolerated in pediatric patients with GHD. In most cases, mean IGF-1 levels remained within the normal range. However, IGF-1 levels may be elevated, particularly in pubertal patients. These findings support somatrogon as a safe and effective early treatment option in routine clinical practice, although larger and longer-term studies are required to confirm long-term safety.

## I**ntroduction**

Growth hormone deficiency (GHD) is a disorder associated with abnormal linear growth, characterized by short stature and reduced age-related growth velocity in children [1]. Since 1985, recombinant growth hormone (rhGH) has been used in the treatment of GHD [2–7]. Growth hormone therapy has been shown to improve linear growth in children with GHD, contribute to the attainment of adult height within the normal range, increase bone mineral density, and improve body composition [8]. Numerous studies have demonstrated that somatropin is effective and safe in the treatment of GHD [2–7]. As reports on the clinical outcomes of patients treated with daily rhGH have accumulated over the years, inadequate treatment adherence has emerged as an important factor negatively affecting optimal growth outcomes [9]. Daily therapy for GHD can represent a substantial burden for both children and their caregivers [10]. Injection-related pain or discomfort [11], medication storage and reconstitution, and disruption of daily activities are among the factors described as treatment-related burden [12]. As observed in multiple studies, these factors complicate adherence to daily subcutaneous injections, leading to missed doses [13] and treatment discontinuation [14]. Long-acting rhGH formulations requiring less frequent administration may help reduce adherence-related challenges associated with daily injections and thereby improve treatment effectiveness [15]. 

Somatrogon is a long-acting growth hormone used in the treatment of GHD [8]. Somatrogon contains the amino acid sequence of human growth hormone (hGH) fused to three copies of the C-terminal peptide (CTP) of human chorionic gonadotropin [8]. In vitro data indicate that somatrogon binds to the growth hormone receptor [8, 16], and animal studies have shown that the addition of CTP to recombinant human growth hormone prolongs its half-life and that somatrogon effectively increases circulating insulin-like growth factor-1 (IGF-1) levels [8]. 

In a multicenter, open-label, randomized phase 2 study of somatrogon, a dose of 0.66 mg/kg/week was shown to result in growth outcomes comparable to those observed in the somatropin group. The annual growth velocity was 11.9 cm in patients receiving somatrogon at a dose of 0.66 mg/kg/week and 12.5 cm in those treated with daily somatropin, demonstrating similar efficacy between the two treatment regimens [17]. Somatrogon was reported to be safe over a 12-month treatment period, with no serious adverse events observed [18]. More recently, two separate phase 3 studies demonstrated that somatrogon administered at a dose of 0.66 mg/kg/week resulted in annual growth velocities of 10.1 cm and 9.6 cm, respectively, and was comparable to daily growth hormone therapy in terms of efficacy and tolerability [8, 12].

Although phase 2 and phase 3 studies in the literature have demonstrated that somatrogon is as effective and well tolerated as daily somatropin, real-world data remain limited. The aim of this study was to evaluate the effectiveness and safety of somatrogon in real-world patients with growth hormone deficiency.

## Materials and methods

### Study population

Patients aged between 3 and 18 years were included in the study. Patients who were followed at our outpatient clinic for short stature, defined as a height below the 3rd percentile accompanied by inadequate growth velocity, underwent routine evaluations to exclude causes other than growth hormone deficiency. After exclusion of these conditions, patients who were diagnosed with growth hormone deficiency based on at least two growth hormone stimulation tests (Clonidine and L-Dopa), with a peak growth hormone level < 10 ng/mL, were included in the study. The levels of other anterior pituitary hormones were evaluated in all cases diagnosed with GHD. In addition, pituitary magnetic resonance imaging (MRI) was performed to exclude intracranial pathology. Patients younger than 3 years or older than 18 years, those diagnosed with Turner syndrome, patients with a history of chronic disease, and children born small for gestational age were excluded from the study.

### Study design and treatment

In this study, the clinical and growth responses of patients who were diagnosed with idiopathic GHD and prescribed somatrogon at the Pediatric Endocrinology Department of Dicle University between January 2024, and October 2025, and who had at least 6 months of follow-up were retrospectively evaluated. The somatrogon dose was initiated at 0.66 mg/kg/week for all patients. Prior to treatment, pubertal status was assessed in all patients according to the Tanner staging system [16]. During the treatment period, patients were scheduled for follow-up visits at 3-month intervals. At each follow-up visit, body weight (BW), body weight standard deviation score (BW SDS), height, height SDS, body mass index (BMI), and BMI SDS were recorded. BW was measured in kilograms using a digital scale with a sensitivity of 0.1 kg (SECA digital scale). Height was measured in the standing position using a Harpenden stadiometer. Height and weight SDS values were calculated according to national reference data using the web-based Child Metrics program [19]. 

At 3-month intervals, along with auxological assessments, thyroid-stimulating hormone (TSH), free thyroxine (fT4), IGF-1, and fasting blood glucose levels were measured. Blood samples were consistently collected on the 4th day (96 h) after somatrogon administration. In addition, at the 6- and 12-month follow-up visits, plasma cortisol and mean HbA1c levels were also measured. Left hand and wrist radiographs were obtained from all patients before treatment initiation and at month 12 for the assessment of bone age. Bone age was determined according to the Greulich–Pyle atlas [20]. During treatment, the somatrogon dose was adjusted at each visit according to the patient’s current BW, serum IGF-1 level, and clinical treatment response. If IGF-1 levels exceeded + 2 SDS at two consecutive visits, the somatrogon dose was reduced by 15%. The primary objective of this study was to evaluate the efficacy and safety of the long-acting growth hormone somatrogon. To assess efficacy, changes in height velocity over a minimum follow-up period of 6 months, height SDS, bone maturation, and IGF-1 levels were evaluated. In terms of safety and tolerability, adverse events as well as clinical, biochemical, and hormonal parameters were assessed.

This study was approved by the Dicle University Medical Faculty Ethics Committee for Non-Interventional Studies (Approval No. 14, November 26, 2025) and was conducted in accordance with the Declaration of Helsinki and the International Conference on Harmonisation Good Clinical Practice guidelines.

### Statistical analyses

Statistical analyses were performed using IBM SPSS Statistics for Windows, version 20.0 (IBM Corp., Armonk, NY, USA). The normality of continuous variables was assessed using the Shapiro–Wilk test. Data with a normal distribution were expressed as mean ± standard deviation (SD), whereas non-normally distributed data were presented as median (25th–75th percentile). Categorical variables were expressed as numbers and percentages (%). Comparisons between pre-treatment and post-treatment measurements were performed using the paired-sample t-test when the assumption of normality was met. A p value < 0.05 was considered statistically significant in all analyses.

## Results

The study included 39 patients with a mean age of 12.90 ± 1.63 years. Of these, 21 (53.8%) were male and 18 (46.2%) were female. Thirty-one patients (79.5%) were pubertal, while 8 (20.5%) were prepubertal. Isolated GHD was diagnosed in all cases. Pituitary MRI findings were normal in all patients.

**Fig. 1:**
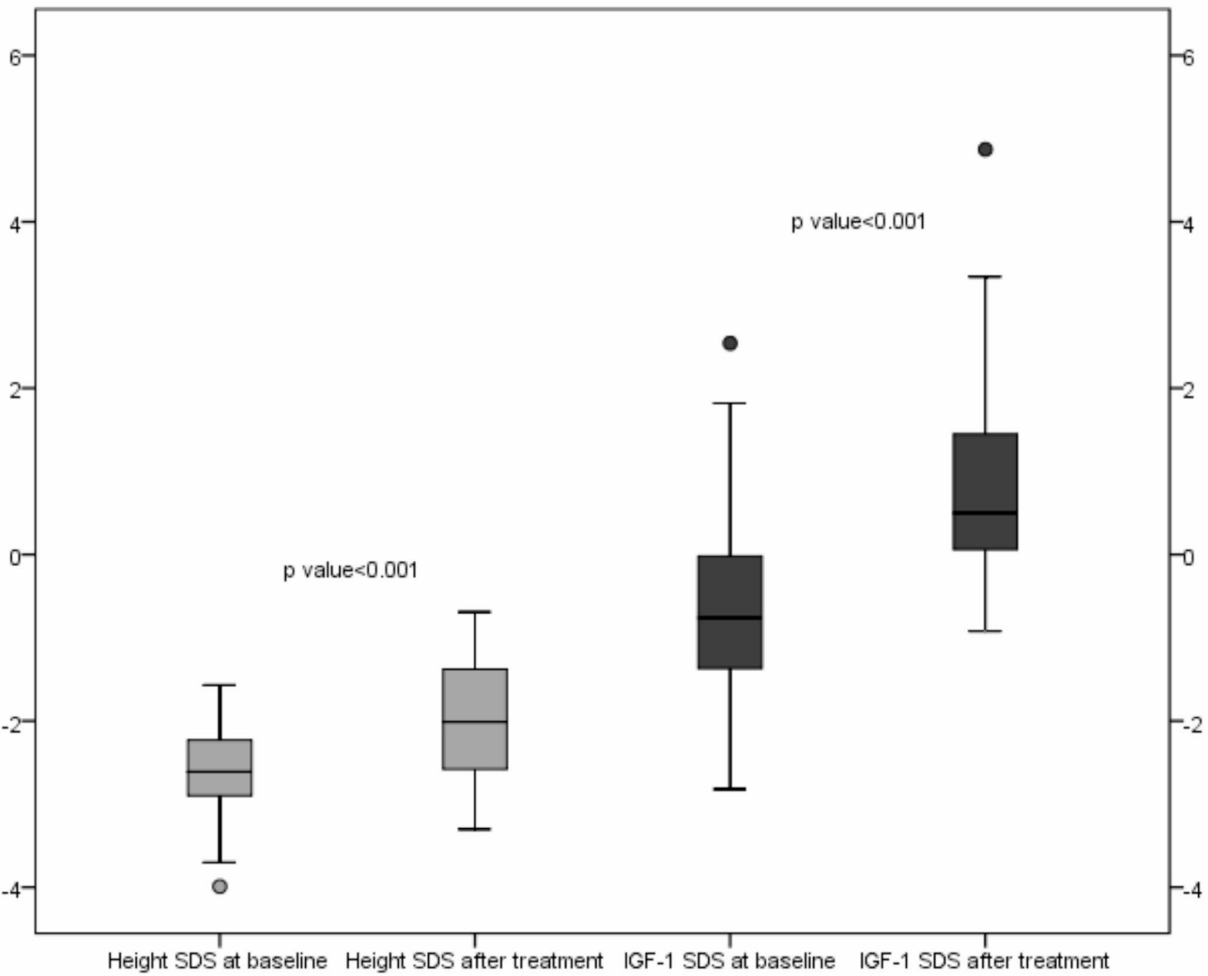
Comparison of Height SDS and IGF-1 SDS at baseline and after the treatment (lines within the boxes indicate the median, the limits of the boxes indicate the 25th and 75th percentiles, and the extensions of the boxes indicate the minimum and maximum)

The mean treatment duration for patients was 11.31 ± 2.72 (min-max: 6.36–16.92) months As this study was designed retrospectively, only patients who had received treatment for at least 6 months were included in the analysis. No patients discontinued treatment during the study period. The mean annual height velocity of patients was determined to be 10.07 ± 0.61 cm/year. Height SDS values at treatment initiation were compared with those at the final follow-up, and a significant increase in height SDS was observed (Δ height SDS: 0.64 ± 0.06; *p* < 0.01) (Fig. 1). Statistically significant improvements were also observed in body weight SDS, height SDS, and BMI SDS values between baseline and the final visit (*p* < 0.01 for all comparisons). No significant change was detected in the bone age to chronological age (BA/CA) ratio between treatment initiation and the final evaluation (0.81 ± 0.10 vs. 0.82 ± 0.08, *p* = 0.2). The pre-treatment and post-treatment anthropometric, laboratory, and radiological characteristics of the cases are summarized in Table 1.

**Table 1:** Comparison of baseline and after-treatment parameters

Variable	At baseline	At after-treatment	*p*
**Age (years)**	12,90 ± 1,63	13,89 ± 1,75	< 0,01
**Body Weight SDS**	-1,84 ± 1,00	-1,13 ± 1,05	< 0,01
**Height SDS**	-2,62 ± 0,53	-1,99 ± 0,66	< 0,01
**BMI SDS**	-0,65 ± 1,10	-0,16 ± 1,03	< 0,01
**Bone Age (years)**	10,49 ± 1,97	11,35 ± 2,00	< 0,01
**BA/CA Ratio**	0,81 ± 0,10	0,82 ± 0,08	0,2
**IGF-1 (ng/mL)**	188,87 ± 85,32	377,75 ± 123,63	< 0,01
**IGF-1 SDS**	-0,61 ± 1,15	0,75 ± 1,26	< 0,01

SDS, standard deviation score; BMI, body mass index; BA, bone age; CA, chronological age; IGF-1, insulin-like growth factor-1

The responses to somatrogon treatment were evaluated according to pubertal status. The Δ height SDS gain was significantly higher in the pubertal group compared to the prepubertal group (*p* = 0.01). Although the annual growth velocity was higher in pubertal cases than in prepubertal cases, the difference was not statistically significant (*p* = 0.41) (Table 2).

**Table 2:** Treatment responses according to pubertal status

Variable	Pubertal (*n*: 31)	Prepubertal (*n*:8)	*p* value
**Baseline Height SDS**	-2,64 ± 0,56	-2,55 ± 0,43	0,69
**Final Height SDS**	-1,93 ± 0,70	-2,21 ± 0,44	0,28
**Treatment duration (years)**	11,60 ± 2,78	10,18 ± 2,27	0,19
**Growth velocity (cm/year)**	10,35 ± 2,84	9,37 ± 3,48	0,41
**Δ Height SDS**	0,70 ± 0,38	0,33 ± 0,30	0,01
**Final IGF-1 SDS**	0,87 ± 1,32	0,16 ± 0,90	0,16

SDS, standard deviation score; IGF-1, insulin-like growth factor-1

The mean IGF-1 level before treatment was 188.87 ± 85.32 ng/mL, which increased to 377.75 ± 123.63 ng/mL at the final follow-up, and this difference was statistically significant (*p* < 0.01). Similarly, the mean IGF-1 SDS at baseline was − 0.61 ± 1.15, whereas the mean IGF-1 SDS at the final visit was 0.75 ± 1.26, representing a statistically significant increase (*p* < 0.01) (Fig. 1). During the study period, 5 of the 39 patients (12.8%) exhibited IGF-1 SDS values > + 2, necessitating a 15% dose reduction. All patients with IGF-1 SDS values > + 2 were in the pubertal stage. In 3 of these 5 patients (60%), IGF-1 SDS levels remained > + 2 despite an initial 15% reduction in the somatrogon dose, and a second dose reduction was therefore required.

### Safety and tolerability

Adverse events reported during treatment were generally mild and transient. Four patients (10.2%) experienced transient headaches that did not require treatment interruption. In these patients, fundus examination was performed to evaluate possible increased intracranial pressure (ICP), and a pediatric neurology consultation was obtained; no findings suggestive of ICP were detected. During follow-up, headache symptoms resolved spontaneously. Transient injection-site erythema was observed in one patient and resolved without intervention. Throughout the study period, no serious adverse events occurred; no lipodystrophy was observed, no deaths were reported, and no patients discontinued treatment due to adverse events.

The mean pre-treatment and post-treatment levels of TSH, free T4, cortisol, and glucose were compared, and no significant differences were found (Table 3). The mean HbA1c level measured during treatment was within the normal range.

**Table 3:** Comparison of laboratory parameters at baseline and after treatment

Parameter	Baseline	After treatment	*p* value
TSH (mIU/mL) (N: 0,79 − 5,85)	2,18 ± 1,03	2,01 ± 0,99	0,33
fT4 (ng/dL) (N: 0,64 − 1,71)	0,86 ± 0,11	0,78 ± 0,26	0,10
Cortisol (µg/dL) (N: 6,7–22,6)	9,60 ± 2,39	9.08 ± 3.66	0,53
Glucose (mg/dL) (N:70–100)	87,21 ± 9,50	85,42 ± 9,52	0,41

TSH, thyroid-stimulating hormone; fT4, free thyroxine, Data presentation: mean ± standard deviation

## Discussion

Somatrogon has been shown to be at least as effective as daily growth hormone therapy in childhood growth hormone deficiency in both phase 2 and phase 3 clinical trials, as well as in real-world studies [8, 12, 18, 21–24]. However, the number of studies addressing real-world data on somatrogon remains limited [23–26]. Therefore, we consider the findings of our study to be valuable. In our cohort of children and adolescents with GHD, somatrogon treatment was associated with an increased growth velocity, a height SDS gain of + 0.64 ± 0.06, absence of serious adverse events in the early treatment period, and good overall tolerability. 

Phase 2 and phase 3 studies on somatrogon have demonstrated that somatrogon is non-inferior to daily somatropin treatment in terms of efficacy [8, 12, 17]. In the 4-year open-label study conducted by Horikawa et al. [22], it was demonstrated that height SDS continued to increase and was similar to daily somatropin. [22]. In a phase 2 study conducted by Zelinska et al., administration of somatrogon at a dose of 0.66 mg/kg/week resulted in growth outcomes comparable to daily somatropin therapy, with no serious adverse events reported and good overall tolerability [17]. In the open-label extension of this cohort continued for up to 5 years. At the end of the fifth year, height SDS had normalized. Additionally, the height SDS gain was shown to be + 3.29 SDS compared to the baseline. Growth responses in this long-term follow-up were comparable to those observed with daily somatropin therapy [21]. Real-world data on the use of somatrogon in children with GHD remain limited. In a recent real-world study involving 39 prepubertal patients, a significant increase in height SDS was observed after 1 year of somatrogon treatment compared with baseline, with a mean height SDS gain of + 0.51 SDS [23]. In a retrospective comparative real-world study reported from Greece, no significant differences were observed between the two groups in terms of height gain and changes in height SDS. [24]. In a recent study conducted in Italy, the Δ height SDS of patients with GHD receiving somatropin treatment were found to be + 0.81 at 18 months. However, this study did not report annual growth velocity [25]. In a real-world study conducted in our country, the annual growth velocity with somatropin therapy was found to be 10 cm, while the Δ height SDS was found to be + 0.6 [26]. In our single-center, retrospective real-world study, patients treated with somatrogon achieved a mean annual height velocity of 10.07 ± 0.61 cm/year and a height SDS gain of + 0.64 ± 0.06. These findings are consistent with both randomized clinical trials and real-world evidence, supporting weekly somatrogon therapy as an effective treatment option in pediatric GHD. 

Randomized clinical trials have demonstrated that weekly somatrogon treatment does not accelerate bone maturation. In the Phase 3 pivotal study, bone age progression at 12 months was reported to be similar between patients receiving somatrogon and those receiving daily somatropin [8]. A Phase 3 randomized study conducted in Japanese children also showed that bone age advancement did not exceed the increase in chronological age [12]. Long-term open-label extension phases of Phase 2 and Phase 3 studies further emphasized that the rate of bone maturation paralleled chronological age advancement [21, 22]. Real-world data also support these findings [23, 24, 26]. In the study by Tamaro et al. [25], it was also shown that the difference between bone age and chronological age remained stable over time during somatrogon treatment. In our study, consistent with previous reports, the BA/CA ratio was similar at baseline and at the final follow-up. These findings suggest that somatrogon therapy supports linear growth while maintaining bone maturation within physiological limits. 

In randomized clinical trials, increases IGF-1 levels have been reported during weekly somatrogon treatment. However, it has been demonstrated that a substantial proportion of this increase is attributable to transient post-injection peaks, as most samples were collected on days 2–3 after administration, reflecting peak IGF-1 levels at these time points. Pharmacokinetic/pharmacodynamic (PK/PD) modeling confirmed that samples obtained approximately 96 h after dose administration represent the mean IGF-1 SDS over the dosing interval. Analyses based on PK/PD modeling showed that the mean IGF-1 SDS largely remained below + 2. Nevertheless, dose reduction was required in approximately 11% of patients due to persistently elevated IGF-1 SDS values on consecutive measurements [8]. In Japanese children, both the phase 3 randomized study and its 4-year open-label extension demonstrated that IGF-1 SDS generally remained within normal limits during somatrogon treatment. However, dose reduction due to consecutive IGF-1 SDS elevations ( > + 2 SDS) was reported in 22.7% of patients during the phase 3 study and in 31% during the open-label extension phase [12, 22]. Similarly, Zelinska et al. reported that mean IGF-1 SDS values generally remained below + 2 in both the phase 2 study and the 5-year open-label extension, and no dose reductions were reported in these studies [17, 21]. In another recent study involving 39 prepubertal patients, somatrogon treatment was associated with a significant increase in IGF-1 levels; however, no information was provided regarding IGF-1 SDS values exceeding + 2 or the need for dose reduction [23]. In a real-world study, dose reduction of 15% was reported in 1 of 10 patients (10%) receiving somatrogon therapy due to IGF-1 SDS levels exceeding + 2 [24]. In one of the two real-world studies published in 2026, IGF-1 SDS was reported to exceed + 2 in 17.5% of cases, whereas in the other study, this proportion was reported as 15.8% [25, 26]. In our study, mean IGF-1 SDS values remained below + 2 SDS during somatrogon treatment while showing a significant increase compared with baseline. Dose reduction of 15% was required in five of 39 patients (12.8%) due to consecutive IGF-1 SDS values exceeding + 2. Notably, all of these patients were in the pubertal stage. In both phase studies and real-world reports, most patients receiving somatrogon treatment were prepubertal [8, 12, 23, 24]. Therefore, closer monitoring of IGF-1 levels may be warranted in pubertal patients receiving somatrogon, and initiation at a lower dose in this subgroup may be considered. 

Previous studies have shown no significant differences in mean glucose levels, HbA1c, or thyroid function tests between patients treated with somatrogon and somatropin therapy [8, 12, 27]. Consistent with the literature, no clinically relevant deterioration in glucose, HbA1c, thyroid function tests, or cortisol levels was observed in our study. These findings suggest that somatrogon therapy does not adversely affect glucose metabolism, thyroid function, or cortisol levels in the short to medium term [8, 12, 27]. 

In the literature, both phase studies and real-world data have shown that somatrogon therapy in children is generally well tolerated, with adverse events typically being mild to moderate in severity [8, 12, 22, 28]. The most frequently reported adverse event is injection-site pain, followed by local reactions such as erythema, pruritus, and induration [8, 12, 22]. Similarly, real-world registry data have reported rare systemic but transient adverse events, including headache and epistaxis [28]. Long-term follow-up studies indicate that serious adverse events are infrequent, no treatment-related deaths have been reported, and discontinuation rates due to adverse events are low [21, 22]. A particularly notable rare adverse effect reported in the literature is injection-site lipoatrophy, which has mainly been described in case reports and small case series [29, 30]. These studies indicate that the primary risk factor for lipoatrophy is repeated injections into the same anatomical site, and that the condition usually resolves completely within a few months when appropriate injection-site rotation is implemented. Proposed pathophysiological mechanisms include the local lipolytic effects of growth hormone, prolonged subcutaneous hormone exposure due to long-acting formulations, and the potential contribution of excipients such as metacresol [29, 30]. National survey–based real-world data also demonstrate that injection-site reactions are the most commonly reported adverse events, whereas lipoatrophy remains a rare occurrence [31]. In our study, adverse events reported during a mean follow-up period of 11.85 ± 0.70 months were mild and transient, consistent with the literature. Four patients (12.8%) developed transient headaches that did not require interruption of treatment. Transient injection-site erythema was observed in one patient and resolved spontaneously. Although injection-site pain was reported by most patients, it was not systematically graded. No cases of lipoatrophy, serious adverse events, deaths, or treatment discontinuations due to adverse events were observed. These findings support the short-term safety of somatrogon therapy.

The main limitations of this study are its retrospective design and the relatively small sample size. The short follow-up period was also one of the limitations of our study.

In conclusion, our study demonstrated that somatrogon therapy in children with GHD resulted in an mean annual height gain of approximately 10 cm, was well tolerated, and was not associated with any serious adverse events. No increase in the BA/CA ratio was observed during the study period. Mean IGF-1 levels remained within the normal range in 87.2% of the patients. We observed that IGF-1 levels may exceed the average particularly in pubertal patients, suggesting that initiation of somatrogon therapy at lower doses may be considered in this subgroup. Overall, our findings support somatrogon as a safe and effective early treatment option in routine clinical practice. However, larger studies with longer follow-up durations are required to confirm its long-term safety.

## Data Availability

The data that support the findings of this study were obtainede from hospital medical records. Due to ethical and privacy consideration, the data are not publicly available.
